# Real-world evidence for the postmarket surveillance of cancer medicines: opportunities and challenges using Australia’s population-based cancer registries

**DOI:** 10.1016/j.esmorw.2026.100748

**Published:** 2026-07-23

**Authors:** B. Daniels, N.S. Meagher, J. Ruiz, C.M. Vajdic, P. Gibbs, S.J. Lord, C. Williams, A. Wilson, N. Pratt, S.-A. Pearson

**Affiliations:** 1 Medicines Intelligence Research Program, School of Population Health, Faculty of Medicine and Health, University of New South Wales (UNSW)–Sydney, Sydney, Australia; 2 The Kirby Institute, University of New South Wales, Sydney, Australia; 3 Personalised Oncology, Walter and Eliza Hall Institute of Medical Research, Melbourne, Australia; 4 Medical Oncology, St Vincent’s Hospital, Melbourne, Australia; 5 The National Health and Medical Research Council (NHMRC) Clinical Trials Centre, The University of Sydney, Camperdown, Australia; 6 The Daffodil Centre, The University of Sydney, and Cancer Council NSW, Sydney, Australia; 7 Leeder Centre for Health Policy, Economics and Data, Faculty of Medicine and Health, University of Sydney, Sydney, Australia; 8 Quality Use of Medicines and Pharmacy Research Centre, School of Pharmacy and Biomedical Sciences, Adelaide University, Adelaide, Australia

**Keywords:** administrative health data, Australia, cancer registry, population-based, postmarket medicine surveillance, real-world evidence

## Abstract

Spending on cancer medicines has increased rapidly worldwide, driven by the introduction of immunotherapy and targeted therapy. Australia exemplifies this trend, with cancer medicines representing one of the largest and fastest-growing areas of expenditure for Australia’s public medicines funder, the Pharmaceutical Benefits Scheme. While this growth reflects therapeutic innovation and expanded clinical use, it has intensified concerns around the real-world safety, effectiveness, and value of novel therapies once adopted into routine care. Randomised clinical trials remain essential for regulatory approval but often provide limited insight into outcomes in broader, more heterogeneous populations, particularly as many therapies enter practice via accelerated pathways based on surrogate endpoints. Population-based cancer registries offer an important resource for postmarket surveillance when linked with national administrative datasets such as dispensing and hospitalisations records. However, limitations in registries’ timeliness, disease stage ascertainment, biomarker and genomic data capture, and information on recurrence and progression constrain their current utility. This perspective examines the Australian population-based cancer registry landscape, highlighting its strengths, untapped potential, and critical gaps. We outline priority enhancements required to realise a robust, whole-of-population cancer medicine surveillance system that can inform clinical practice, policy, and sustainable health care decision making.

## Highlights

• Australian population-based cancer registries enable real-world evidence on medicine use and outcomes.
• Data gaps in disease stage, biomarkers, and recurrence limit registry potential.
• Robust medicine surveillance requires timely data, clinical detail, and governance.
• We examine the opportunities and limitations of Australian cancer registries.

Worldwide, spending on cancer medicines has risen sharply over the past decade, driven primarily by the rapid expansion of immunotherapy and targeted therapy.^1^ Australia exemplifies this phenomenon, where cancer medicines represent one of the largest- and fastest-growing areas of expenditure under the nation’s Pharmaceutical Benefits Scheme (PBS).

As expenditure has grown, so too have concerns from payers, clinicians, and patients around the real-world effectiveness, safety, and overall value of cancer therapies. Randomised clinical trials (RCTs) are the gold standard for demonstrating the short-term safety and efficacy of new cancer medicines, but trial populations and the tightly controlled treatment protocols followed in RCTs can differ substantially from those encountered in routine care.^2^ Further, over the past decade, many new therapies entered clinical practice through accelerated approval pathways, often based on surrogate endpoints or single-arm trials.^3^^,^^4^ With many treatments now in use across multiple indications and lines of therapy, real-world evidence on medicine safety and effectiveness from robust postmarket surveillance is seen as increasingly important to ensure the risk-to-benefit ratios demonstrated in RCTs are realised in the community.

Population-based cancer registries capturing diagnosed cancers offer a powerful foundation for medicine surveillance. However, their utility for generating high-quality evidence depends on their timeliness, completeness, granularity, and capacity for linkage with other population-based datasets containing information on cancer treatments and outcomes. Australia’s evolving data landscape across these domains uniquely positions its researchers to contribute to real-world evidence generation, but important gaps remain. In this perspective, we examine the current landscape of Australian cancer registries and their potential to generate real-world evidence on publicly funded cancer therapies. We outline the critical enhancements required to realise this potential and explore the key barriers that must be addressed for registries to meaningfully inform clinical practice, policy, and system performance.

## High-quality australian cancer registries: A strong foundation

Cancer, other than nonmelanoma skin cancer, is a notifiable disease in Australia, with each of its six states and two territories legally mandated to maintain a central register of primary cancer diagnoses. These registries were established predominantly to monitor cancer incidence and mortality rates and to support health system management, service planning, and funding decisions.^5^ While there is typically a lag between the current day and the most recent registry diagnoses available to researchers (generally 1-3 years), the near-complete population coverage of cancers diagnosed is a major strength of population-based registries as opposed to hospital- or pathology-based registries, which capture patients from selected hospitals or specific patient groups.^6^

Australian state/territory population-based cancer registries collect a core set of measures including date and basis of diagnosis, tumour topography and morphology, sex, date of birth, and postcode and have been routinely linked with Australia’s National Death Index to obtain date and cause of death information since the 1990s. These measures, common across the states and territories, are consolidated into the Australian Cancer Database (ACD), providing national-level statistics and support for approved research.^7^

While the completeness of data for the core set of measures comprising the ACD is high^8^—critical for population-level investigations—individual state/territory cancer registry data can vary substantially in terms of additional, if not essential, clinical indicators. For example, the cancer registry in New South Wales, Australia’s most populous state, records high-level information about the extent of disease spread at diagnosis (localised, regional, or distant),^9^ which may be used as a surrogate for disease stage. Some registries, including the New South Wales registry, include American Joint Committee on Cancer TNM (tumour–node–metastasis) staging information derived from data not included in the registry (but available to the data custodians). This information is typically only available for specific years and for specific cancers.^10^ Similarly, some registries endeavour to collect specific biomarker information where such information is routinely captured in structured pathology reports (e.g. HER2 status), but completeness of these measures is often low in practice. These gaps limit the ability to stratify outcomes by tumour stage and molecular subtype, an increasingly important requirement for evaluating contemporary cancer medicines that are based on molecular phenotypes.

## Linking registries with national health data: Strengths and blind spots

The value of cancer registries for postmarket medicine surveillance has been expanded substantially through linkage with national administrative health datasets, including PBS dispensing and hospital admissions. Australia operates a multilevel, publicly funded health care system, ensuring all citizens and permanent residents receive subsidised hospital care, medical services, and cancer treatments regardless of income. In this arrangement, the federal government subsidises medicines dispensed in the community and private hospitals through the PBS, the state and territory governments pay for public hospital care. As a result, states and territories are incentivised to administer the overwhelming majority of cancer medicines to patients in ‘outpatient’ care where it is funded by PBS, shifting the cost of these medicines to the federal government. Additionally, once a medicine is subsidised by PBS, private insurers will not provide any reimbursement for that medicine and specific indication for which it is PBS listed.

While some patients may access new cancer medicines through clinical trials or, before subsidies are approved, via compassionate access schemes or private health insurance, Australia’s publicly funded medicine program has created a situation where the overwhelming majority of cancer medicines administered as part of routine care in Australia are captured within PBS dispensing data.^11^ As a consequence, when linked to cancer registry data, PBS data are generalisable from a clinical and payer perspective, facilitating investigations of complex exposure regimens, the use of treatments in different settings (e.g. first- or second-line treatment), and treatment switching and adherence, all within the actual population treated in routine care. Gaps in data due to patients accessing treatments outside of the PBS are small but likely to disproportionately comprise early adopters of new therapies, patients with rare cancers, and those accessing innovative medicines before public reimbursement. Improving the capture of non-PBS therapies will become increasingly important as new medicines are accessed under expedited regulatory and reimbursement pathways in areas of high unmet clinical need, such as rare disease.

## Beyond survival outcomes

Apart from mortality data, cancer registries do not routinely capture outcomes critical to assessing the safety and effectiveness of cancer treatments, such as disease relapse and progression over time. However, linkage with Australia’s rich hospital admissions data facilitates the ascertainment of proxy measures indicative of real-world effectiveness. For instance, an admission record for a major cardiac event following treatment with a specific agent may indicate a treatment-related safety signal. Additionally, diagnosis codes from hospital admission records, paired with dispensing records through further linkage with PBS data, may indicate disease recurrence or progression to metastatic disease.^12^^,^^13^ While the validity of proxy outcomes cannot be assumed and substantial methodological work is required to ensure the derived endpoints are valid in terms of their clinical meaning, linkage with other Australian population-based, routinely collected administrative health datasets provides opportunities to further augment the research value of cancer registries.

## National data infrastructure: Momentum and opportunity

**Figure 1:**
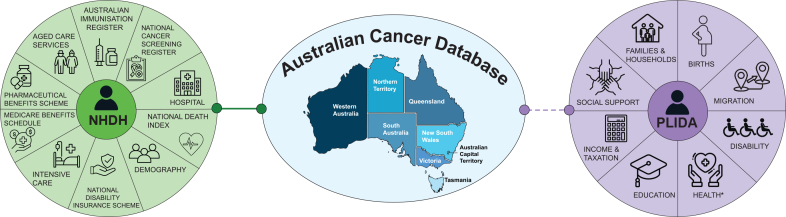
**Linked data infrastructure supporting cancer research in Australia at the population level.** The National Health Data Hub (NHDH) and the Australian Cancer Database (ACD) are managed by The Australian Institute of Health and Welfare. The Person-Level Integrated Data Asset (PLIDA) is hosted by the Australian Bureau of Statistics. The ACD is currently linked to the NHDH (solid line); future linkage between the ACD with PLIDA is planned (dashed line). ∗Health comprises multiple datasets including the Pharmaceutical Benefits Scheme, Medicare Benefits Schedule, Australian Immunisation Register, National Cancer Screening Register, and National Death Index, among others.

In recent years, Australia has made substantial investments in large-scale national data infrastructure. Both the Australian Institute of Health and Welfare (AIHW) and the Australian Bureau of Statistics (ABS) now host integrated, person-level linked data assets (Figure 1). The AIHW’s National Health Data Hub^14^ has recently incorporated cancer registry data from multiple jurisdictions into a national spine linking PBS and hospital records. The ABS-hosted Person-Level Integrated Data Asset^15^ further enables enduring linkage with socioeconomic data, including education and employment, supporting equity-focused analyses of cancer care and outcomes.

Continued harmonisation of these assets will enable not only timelier postmarket surveillance of cancer medicines but also more nuanced analyses of use and outcomes in underserved populations. The 2025 National Cancer Data Framework underscores this opportunity, identifying streamlined governance, improved data capture through harmonisation, and strengthened reporting frameworks as priorities for building a world-class cancer medicine surveillance system.^16^

## The future is bright, but action is required

Australia’s emerging data infrastructure is well positioned to support substantial global contributions to real-world cancer medicine surveillance, on par with existing national data resources such as England’s National Cancer Registration and Analysis Service and Systemic Anticancer Therapy dataset, as well as the Nordic countries’ NORDCAN.^17^, ^18^, ^19^ To further upscale our capacity to transform postmarket cancer medicine surveillance we need to prioritise the following: (i) reducing the lag time of cancer registrations, enabling more timely analyses within the context of the rapidly moving cancer medicines landscape; (ii) finding novel, validated ways to increase the timeliness and efficiency of systematic enhanced data capture, for example, the use of artificial intelligence/natural language processing taking advantage of structured pathology reporting and routine tumour molecular profiling; (iii) leveraging robust data standard models, such as the Observational Medical Outcomes Partnership Common Data Model, which is widely used to standardise data and facilitate large cross-jurisdictional studies [see, e.g. the Australian Health Data Evidence Network (AHDEN) and Data Analysis and Real World Interrogation Network (DARWIN EU) initiatives]^20^, ^21^, ^22^; (iv) validations of outcome proxy measures; and (v) code sharing to capitalise on the rich sources of health information enabling data linkage capabilities.

## Conclusion

Australia’s population-based cancer registries are a national asset, offering near-complete coverage of incident cancers and a strong foundation for impactful real-world evidence generation. When linked with other population-based datasets, these registries provide a powerful platform for evaluating the safety, effectiveness, and value of cancer medicines in routine practice. Nonetheless, limitations in ascertainment of cancer stage, biomarker, recurrence and progression data, treatment details, and timely outcome data constrain their full potential for postmarket medicine surveillance. With continued investment and coordinated national effort, Australia is poised to build one of the world’s most robust systems for monitoring cancer medicine outcomes at a whole-of-population level—supporting payers, clinicians, and patients in this era of rapidly evolving and increasingly costly cancer therapeutics.
